# Knockout of *CLTC* gene reduces but not completely block SFTSV infection

**DOI:** 10.1371/journal.pone.0285673

**Published:** 2023-08-25

**Authors:** Tiezhu Liu, Jiajia Li, Xueqi Wang, Tao Huang, Wei Wu, Aqian Li, Chuan Li, Xiaoxia Huang, Qin Wang, Dexin Li, Shiwen Wang, Mifang Liang

**Affiliations:** 1 National Health Commission Key Laboratory for Medical Virology, National Institute for Viral Disease Control and Prevention, Chinese Center for Disease Control and Prevention, Beijing, China; 2 The First Affiliated Hospital of Anhui Medical University, Hefei, China; 3 Capital Institute of Pediatrics, Beijing, China; University of South Florida, UNITED STATES

## Abstract

Clathrin is a key protein for viruses to enter host cells. Previous studies often use clathrin inhibitors or gene knockdown technology to partially inhibit the function of clathrin, but whether SFTSV can infect host cells without clathrin expression remains unclear. In this research, a clathrin heavy chains (CLTC) knockout A549 cell line was established by CRISPR/Cas9 technology, and the knockout of *CLTC* was verified by PCR, Western blot, immunofluorescence and T7E1 analysis. The off-target effect was evaluated by PCR combined with Sanger sequencing. Furthermore, this research verified that SFTSV infection was significantly inhibited, but not completely blocked, due to the deletion of CLTC protein. Our research also found that lipid raft inhibitor Filipin, other than macropinocytosis inhibitor EIPA, could significantly reduce SFTSV infection, and the inhibition was more obviously observed when Filipin was used in *CLTC* knockout cells. These result indicated that clathrin-dependent and lipid raft mediated endocytosis are the major two mode used by SFTSV entry. In conclusion, this study constructed a *CLTC* knockout cell line, which, for the first time, established a cell model for the study of the function of CLTC protein, and provided direct evidence that SFTSV pendent could still infect cells without clathrin. Additionally, we confirmed that lipid raft mediated endocytosis, as a clathrin-independent pathway, could be another key mode for SFTSV entry.

## Introduction

Clathrin-mediated endocytosis (CME) is the major endocytic pathway in mammalian cells [1]. It is also responsible for the entry of viruses and other pathogens into host cells [2]. CME occurs via the assembly and maturation of clathrin-coated pits (CCP) that concentrate cargo as they invaginate and pinch off to form clathrin-coated vesicles (CCV) [3]. Clathrin molecules, as the major coat proteins of CCP and CCV, have a triskelion structure composed of three noncovalently bound clathrin heavy chains (CLTC) which provide the structural skeleton of clathrin lattice, and three light chains which regulate the formation and decomposition of clathrin lattice [4]. Clathrin is not only crucial in the formation of CCV, but also plays an important role in virus endosomal transport [5].

Severe fever with thrombocytopenia syndrome virus (SFTSV), a novel highly pathogenic bunyavirus, was first discovered in the Dabie Mountains in China [6] and causes severe fever with thrombocytopenia syndrome (SFTS) with an initial case fatality rate of 30%. SFTSV infections have been reported in over 26 provinces in China [7], Japan [8], and South Korea [9]. Similar viruses have also been reported in the US [10]. Currently, due to the lack of study on the mechanism of SFTSV infection, there are still no vaccines or specific antivirals available against SFTSV infection.

The classical endocytic mechanisms for viruses mainly include clathrin-dependent endocytosis, lipid raft-mediated endocytosis, and micropinocytosis [11]. A previous study showed that inhibitors of dynamin [12] caused a significant decrease of SFTSV infection, and another study using quantum dots (QDs) based single particle found that SFTSV infection led to recruitment of clathrin onto the cell surface [13], both suggesting that the entry of SFTSV may be clathrin dependent. However, due to the lack of direct evidence, it was not clear that whether SFTSV can enter host cells without clathrin expression. Therefore, construction of *CLTC* gene knockout cell line will help to verify this hypothesis so that to deeply analyze the mechanism of endocytosis and provide theoretical support for the discovery of drug targets for SFTSV.

CRISPR/Cas9 technology, allowing specific DNA editing of targeted genes by RNA-directed Cas9 nuclease with high efficiency of gene knockout, has been applied to various species as the most widely used gene editing technology [14–17]. Until now, it has not been reported to establish clathrin knockout cell lines, or to use clathrin knockout cell line to study the functions of clathrin during virus infections. In this study, a *CLTC* gene knockout cell line was constructed by CRISPR/Cas9 system combined with lentivirus packaging technology, and the knockout efficiency was confirmed in both DNA and protein levels, and then, the effect of this gene knockout on SFTSV infection was evaluated, which will lay a solid foundation for the future study of clathrin in SFTSV infection.

## Materials and methods

### Cells and viruses

Cell lines (A549, HeLa, Vero and HEK293FT cells) were initially acquired from the American Type Culture Collection (ATCC; USA), and were cultured at 37°C under 5% CO_2_ in Dulbecco’s modified Eagle’s medium (DMEM; Life Technologies, USA) supplemented with 10% heat-inactivated fetal bovine serum (FBS; Life Technologies, USA) and 1% penicillin/streptomycin (PS, Life Technologies, USA). Cells were passaged every 2 days and digested with 0.05% trypsin–EDTA.

SFTSV strain HB29 was first isolated in our laboratory [6], then passaged in Vero cells as a virus stock, and further utilized to infect A549 cells at an MOI of 0.1 for the gene knockout validation experiments.

### Design and synthesis of sgRNA

*CLTC* gene sequence was searched on GeneBank to determine the distribution of exon region. On the basis of the second exon sequence, the online tool (http://crispr.mit.edu/) was used to design the sgRNA(single guide RNA) sequence. CACC was added to the 5’ end of the sequence. AAAC was added to the 5’ end of the complementary sequence. The sequences were synthesized by RuiBiotech (Beijing, China). The synthesized sgRNA primers were phosphorylated and annealed to form a sgRNA duplex for further study.

### Construction of lentiCRISPR v2-sgRNA expression vector

The lentiCRISPR v2 (Addgene No. 52961) was digested by Esp3i, and sticky ends were obtained. The reaction system was as follows: Nuclease-free water, 14μL; 10×FastDigest Green Buffer, 2μL; DTT (20mM), 1μL; lentiCRISPR v2, 2μL; FastDigest enzyme, 1μL, followed by an incubation at 37°C for 5 min。

The product was electrophoresed on a 1% agarose gel and the digested product was recovered. The phosphorylated and annealed sgRNA was ligated to the recovered digested product by a rapid ligase. The ligation product was transferred to ss320 competent cells (Biorise, China), and the clones were screened on ampicillin-resistant LB plates. The positive clones were screened and sequenced by Ruibiotech (Beijing, China). The recombinant plasmid was extracted from the correct clones.

### Lentivirus packaging

HEK293FT cells were seeded into 6-well plates, and when the cell density reached 80%, supernatant was sucked off, and 1mL of pre-incubated OPTI-MEM medium was added to each well. Using the Lipofectamine 3000 Transfection Reagent Kit (Invitrogen, USA), The constructed lentiCRISPR v2-sgRNA plasmid and two other lentiviral packaging plasmids psPAX2 (Addgene No. 12260) and pMD2.G (Addgene No. 12259) were co-transfected into HEK293FT cells at a molar ratio of 2:1:1. At 60h after transfection, the lentivirus was harvested and centrifuged in 1.5mL eppendorf tube at 15000×g at 4°C for 5 min. The supernatant was retained and filtered with a 0.45μm filter before being aliquoted and frozen at -80°C.

### Acquisition of monoclonal cells

A549 cells were infected with the packaged lentivirus at MOI = 0.2. The next day, 2μg/mL puromycin was added to the cell supernatant for drug screening. Four days later, A549 cells integrated with lentivirus were obtained and were diluted and seeded into 96-well plates with 1 cell per well. After the single cell grew into a cell mass, the monoclonal cell mass was digested and moved to a 6-well plate to continue the culture.

### T7E1 analysis for the knockout efficiency of *CLTC* gene

GeneArt^®^ Genomic Cleavage Detection Kit (Life Technology, USA) was used to evaluate the knockout effieciency. Loci where the gene-specific double-strand breaks occur was amplified by PCR. The PCR product is denatured and reannealed so that mismatches are generated as strands with an indel re-annealed to strands with no indel or a different indel. Then, samples were treated with and without T7 endonuclease 1(T7E1) and the resultant bands are analyzed by gel electrophoresis. A PCR template provided by the kit was used as a positive control.

### PCR verification of *CLTC* gene knockout

DNA was extracted from monoclonal cells and processed PCR amplification with a forward primer sequence of 5’-GGTGGTAATCATTGATATGA-3’, and a reverse primer sequence of 5’-GAATCCAGCTAGCAAAGTA-3’. The PCR reaction condition was as follows: 40 cycles including 95°C for 5 min, 95°C for 30 seconds, 60°C for 30 seconds and 72°C for 30 seconds, followed by a final extension at 72°C for 10 min. The PCR product was then subjected to Sanger sequencing.

### Western blotting analysis for the verification of *CLTC* gene knockout

Expression of CLTC protein in the sgRNA control, *CLTC* knockdown and *CLTC* knockout cells was subjected to Western blotting analysis. Cells were rinsed once with ice-cold PBS, trypsinized, resuspended in PBS, and lysed by RIPA regent, and the supernatant was centrifuged. After the sample protein concentration was determined, an equal amount of protein sample was subjected for sodium dodecyl sulfate-polyacrylamide gel electrophoresis (SDS-PAGE). Separated proteins were transferred to a polyvinylidene difluoride (PVDF) membrane (Bio-Rad, USA) which had been blocked with 5% skim milk in PBS for 1h. The membrane was incubated overnight at 4°C with a rabbit polyclonal antibody directed against CLTC (Abcam, USA), and then washed three times with PBST [18]. β-actin detected with rabbit anti-β-actin antibody (Cell Signaling Technology, USA)was used as loading control. The membrane was then incubated with an HRP-conjugated secondary antibody for 1h and washed three times with PBST for 10 min each [18].

### Immunofluorescence analysis for the verification of *CLTC* gene knockout

For preparation for Immunofluorescence analysis, A549 cells were loaded into 8-well glass slides until an 80% confluence. Then cells were fixed with 4% paraformaldehyde and permeabilized with 0.25% Triton X-100 for 30 min at room temperature and blocked with PBS containing 3% bovine serum albumin for 1h. Then, cells were stained overnight at 4°C with a rabbit polyclonal antibody against CLTC (Abcam, USA) and an anti-rabbit IgG antibody conjugated with Alexa Fluor 488 (Invitrogen, USA) was utilized as the secondary antibody [18].

### Evaluation of off-target effect and cell proliferation activity detection

According to the designed sgRNA sequence, the online off-target effect prediction tool (CRISPOR) was used to obtain the top 10 sites that were most likely to be off-target. The 10 sites were amplified by PCR and sequenced. BioEdit software was used to compare the amplified sequence with the wild-type gene sequence to verify the existence of variation.

The wild-type cells and *CLTC* gene knockout cells were seeded into a 96-well plate with 5 replicates for each kind of cells. Cell proliferation detection reagent MTT Cell Assay Kit (Biotium, USA) was added to the cell supernatant and the absorbance value was detected at 570nm by a Multiskan SkyHigh microplateReader (Thermo Fisher, USA). The OD570 values of the two kinds of cells were compared to determine whether there was a difference in the proliferation activity of the two kinds of cells.

### SFTSV infection essay and detection of replication kinetic of SFTSV

A549 cells of wild-type and *CLTC* knockout were seeded into 6-well plates, followed by SFTSV infection with MOI = 0.1, cells infected with SFTSV and the supernants were collected at 0h, 24h, 36h and 48h after infection. RT-PCR was used to detect the viral RNA in the cells at four time points as described above. Ratios of log (viral RNA copies) of tested samples to those of GADPH were used to compare relative levels of RNA levels of SFTSV. The error bars represent the means ± standard deviation with 95% confidence interval (n = 10 each). The *p* value was calculated by the unpaired two-tailed t-test.

ELISA(Enzyme-Linked Immunosorbent Assay) and Western blotting analysis were used to detect the expression of SFTSV nucleoprotein in the supernant and in the cells, respectively. For ELISA analysis, 96-well plates were coated with a rabbit polyclonal antibody directed against SFTSV (500ng per well); Culture supernatants, which were diluted by 1:1 and 1:10 respectively, together with mixture of TritonX-100 and NP40 reagent, were added to 96-well plates followed by an incubation at 37°C for 1 h. Horseradish peroxidase (HRP)-labeled mouse monoclonal antibody against SFTSV nucleoprotein was added, followed by an incubation at 37°C for 1 h. Then, plates were washed five times, and substrate tetramethylbenzidine (TMB) in citrate buffer was added to each well. The addition of 2 NH_2_SO_4_ stopped the reactions, then optical densities (at 450 nm) were determined with an ELISA reader (Thermo Scientific, USA) [18]. The lower limit of positivity(cut-off) was deemed to be the mean OD plus two standard deviations of the three negative control samples. Ratios of OD450 value of tested samples to mean value of OD450 of negative controls were used to compare relative levels of nucleoproteins of SFTSV.

For Western blotting analysis, cells were collected in PBS, centrifuged and resuspended in RIPA buffer. Equal amount of each sample was fractionated by 10% SDS-PAGE gel. Separated proteins were transferred to a polyvinylidene difluoride (PVDF) membrane (Bio-Rad, USA) followed by blockage with 3% bovine serum albumin, incubated overnight at 4°C with a mouse antibody directed against SFTSV nucleoprotein, and an anti-mouse second antibody was added, followed by incubation with an HRP-conjugated secondary antibody for 1 h and washed three times [18].

Filipin(Biovision, USA), a lipid raft inhibitor, and EIPA(Apexbio, USA) a macropinocytosis inhibitor, were added to cell culture supernatant, respectively, followed by incubation at 37°C for 4 h and rinsed with PBS for three times before SFTSV infection, according to manufacturer’s instruction.

### Confocal microscopy analysis

For preparation for confocal microscopy, *CLTC* knockout and the wild-type cells were loaded into 8-well glass slides and infected with SFTSV at an MOI of 100 after they had grown to 80% confluence. Then, cells were fixed with 4% paraformaldehyde at 36 h post infection. Then, cells were permeabilized with 0.25% Triton X-100 for 30 min at room temperature and blocked with PBS containing 3% bovine serum albumin for 1 h at room temperature. Cells were then stained overnight at 4°C with a mouse monoclonal antibody targeted at SFTSV GP [18], then stained with a secondary anti-mouse IgG antibody conjugated with Alexa Fluor 488 for 30 min at room temperature. Finally, cells were observed with a Leica TCS SP8 laser scanning confocal microscope (Leica, Germany).

## Results

### Construction and identification of recombinant expression vector LentiCRISPR-sgRNA

**Fig 1:**
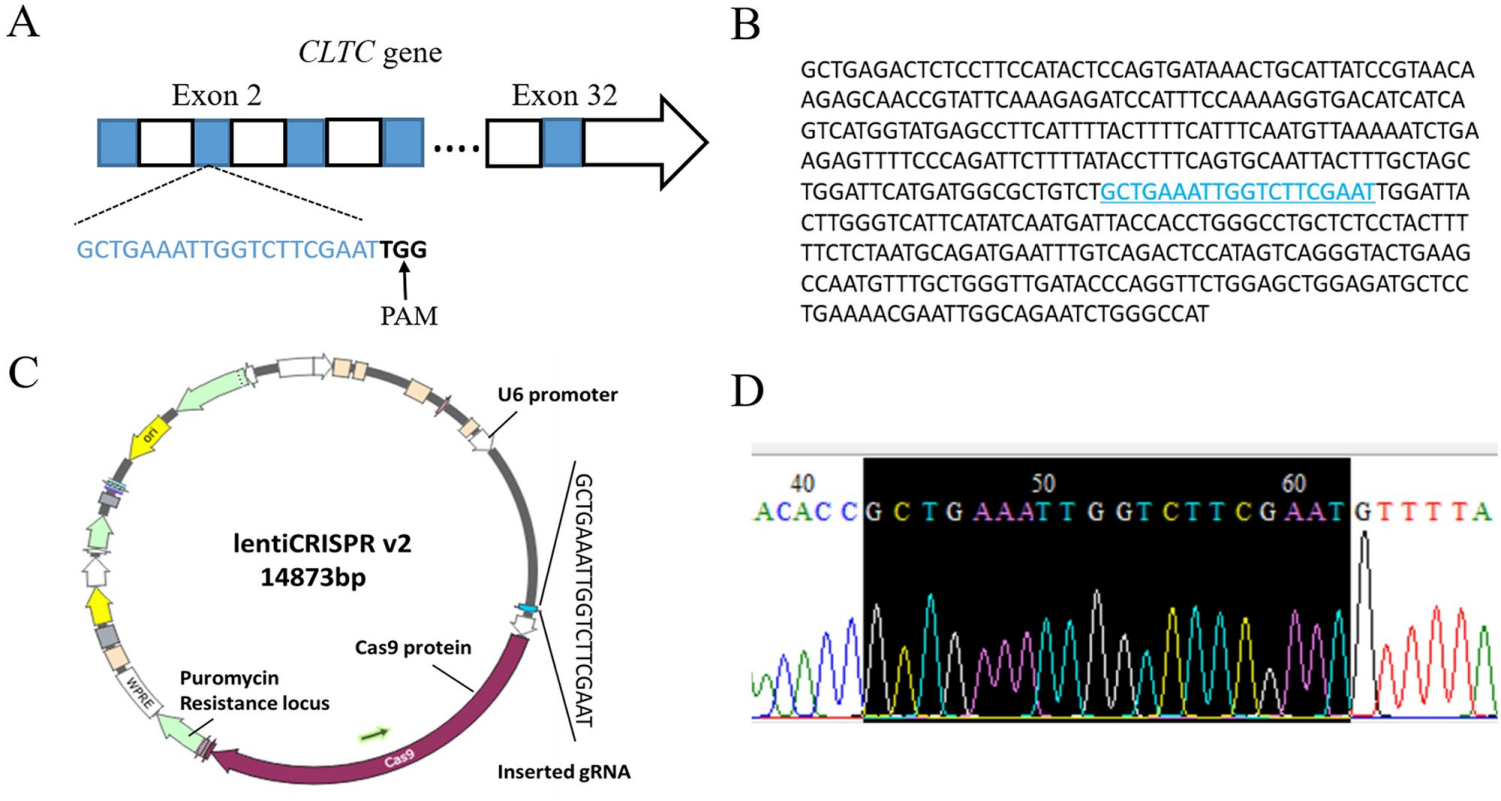
*CLTC* sgRNA targeted site and construction of lentiCRISPR-sgRNA vector. **A** Schematic diagram of *CLTC* sgRNA targeting location. Blue boxes indicates exons of *CLTC* gene, Blue font indicates the sgRNA sequence followed by a PAM sequence of TGG. **B** Sequence of the sgRNA targeting exon, Blue font indicates the exact location of sgRNA targeting. **C** Schematic diagram of the structure of LentiCRISPR-sgRNA. **D** Identification of the insertion of *CLTC*-sgRNA into lentiCRISPR v2 vector by Sanger sequencing. Sequence with a black background represents the inserted sgRNA.

There are totally 32 exons in coding region of *CLTC* gene, and the sgRNA sequence of GCTGAAATTGGTCTTCGAAT followed by a PAM (Protospacer adjacent motif) sequence of TGG was obtained using the online tool according to the second exons (Fig 1A). The specific targeting position were shown in Fig 1B. The designed sgRNA sequence was then cloned into the lentiCRISPR v2 plasmid with a puromycin resistance and Cas9 gene, and the vector information and cloning position were shown in Fig 1C. Finally, constructed plasmid was subjected to Sanger sequencing, which showed the sgRNA sequence inserted was completely consistent with the designed sequence(Fig 1D). These data indicated the fragment was cloned successfully.

### Generation and verification of *CLTC* gene knockout cell lines

A total of 7 monoclonal cell lines were obtained from a 96-well plate, and expanded to 6-well plates. Genomic DNA was extracted, and fragments containing sgRNA targeting sequences were amplified by PCR and subjected for Sanger sequencing, and 4 monoclonal cell lines among 7 were detected to be wild-type cells.

**Fig 2:**
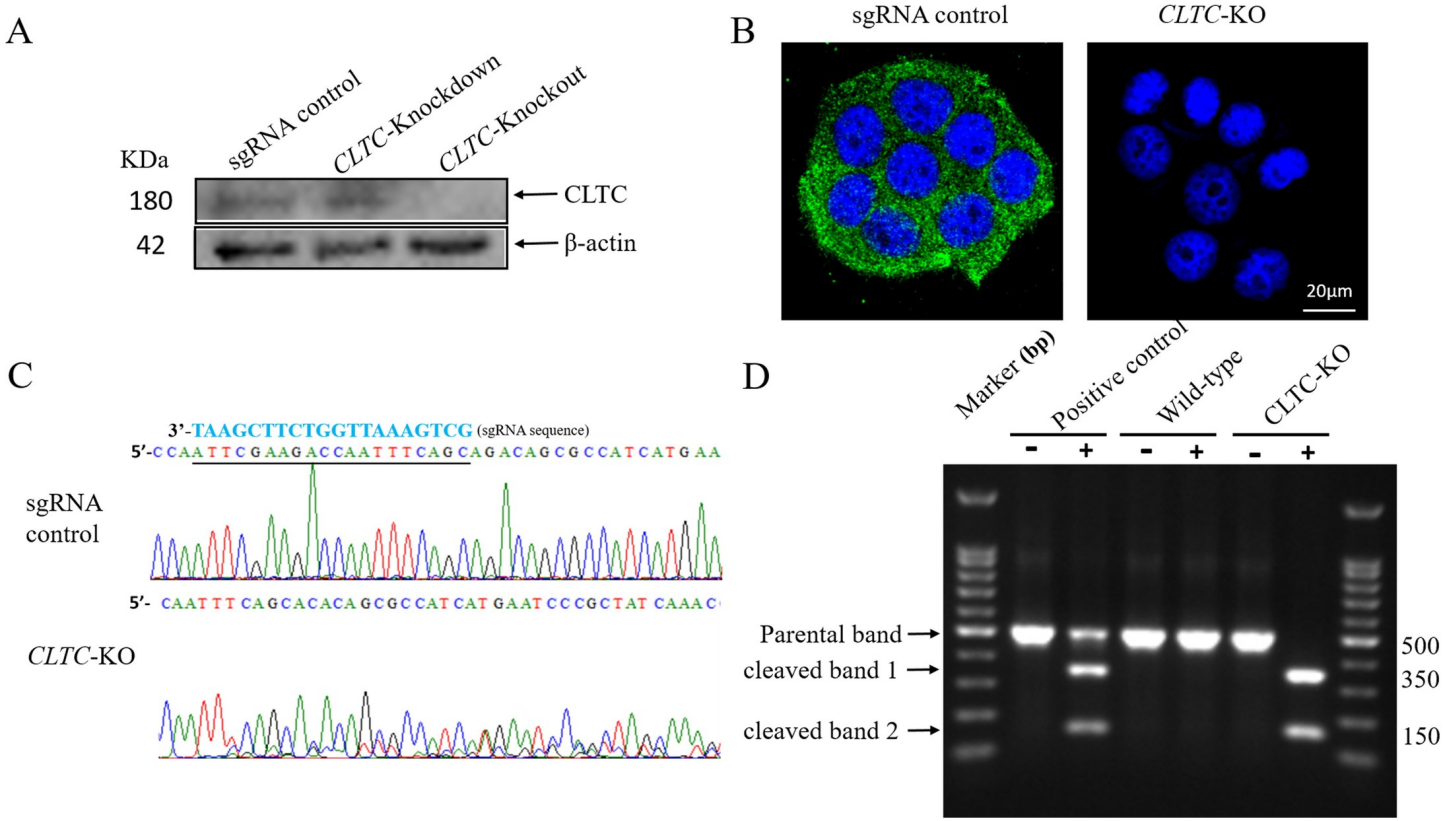
Verification of *CLTC* gene knockout in genetic and protein levels. **A** Verification of *CLTC* gene knockout by western blotting analysis using sgRNA control, *CLTC* knockout and *CLTC* knockdown cells. β-actin was used as the loading control. **B** Verification of *CLTC* gene knockout by Immunofluorescence assay. **C** Verification of *CLTC* gene knockout by Sanger sequencing. *CLTC*-KO represents *CLTC* gene knockout cell line. **D** T7E1 essay for the knockout efficiency. Loci where the gene-specific double-strand breaks occur was amplified by PCR. The product is denatured and reannealed so that mismatches are generated. Then, samples were treated with and without T7 endonuclease 1(T7E1) and the resultant bands are analyzed by gel electrophoresis. A PCR template provided by the kit was used as a positive control.

Among the other 3 cell lines whose targeted sequences were interfered, only one cell line showed no CLTC protein expression and one showed decreased expression by western blotting analysis(Fig 2A). The *CLTC* knockout cell line was also verified by Immunofluorescence analysis (Fig 2B). Sanger sequencing results of *CLTC* knockout cell line showed there were over-lap peaks after the targeted sequence, and the base sequences after the targeted sequence were completely different from the sgRNA control cells (Fig 2C). Since there was no CLTC protein expression in the *CLTC* knockout cell line, our results demonstrated that the *CLTC* genes in the two homologous chromosomes were both disrupted by sgRNA guided Cas9 nuclease with different repair mode between the two homologous chromosomes, leading to the appearance of the over-lap peaks. In general, the *CLTC* gene knockout cell line was successfully constructed and verified.

To better understand the knockout efficiency in our research, we performed a T7E1 assay. The targeted sites where the gene-specific double-strand breaks occur are amplified by PCR. The mismatches are subsequently detected and cleaved by T7 endonuclease 1 and then the resultant bands are analyzed by gel electrophoresis. The result showed that there was only one obvious parental band(500bp) in sgRNA control cells when T7 endonuclease 1 was added, while there were no parental bands but two cleaved bands(350bp and 150bp) in *CLTC* knockout cells, as shown in Fig 2D, which indicated that the *CLTC* gene was successfully knocked out in this research.

### Evaluation of off-target effects and cell proliferation activity

The sgRNA sequence was input to CRISPOR website to obtain the off-target sites that may be caused by this sgRNA. The top 10 sites that are most likely to have off-target effects were selected, and their accession number, off-target score and mismatch positions were listed in Table 1.

**Table 1:** Top 10 off-target sites of the designed sgRNA sequence.

ID number	Off-target sequences	Mismatch position	Off-target score	Chromosome
0ff-taget-1	GCTGAAGTAGGCCTTCAAATGGG	......*.*..*....*...	0.5795	chr2
0ff-taget-2	GTTGATATTTGTCTTCAAATTGG	.*...*...*......*...	0.4924	chr2
0ff-taget-3	GATGAAACTGGTCATAGAATTGG	.*.....*.....*.*....	0.4502	chr6
0ff-taget-4	GCAGAAATTAGTTTTCAAATCGG	..*......*..*...*...	0.4356	chr9
0ff-taget-5	TCTGAAATTGGTTTAAGAATGGG	*...........*.**....	0.4053	chr17
0ff-taget-6	GTTGAAGTTGGGCTTAGAATTGG	.*....*....*...*....	0.3209	chr17
0ff-taget-7	CCTGAAATGGGTTTTAGAATAGG	*.......*...*..*....	0.3100	chr18
0ff-taget-8	TCACATATTGGTCTTCGAATTGG	*.**.*..............	0.2701	chr2
0ff-taget-9	GCTTAGATTAGTCTTAGAATTGG	...*.*...*.....*....	0.2424	chr2
0ff-taget-10	GCTGAAAAAGGGCATCGAATGGG	.......**..*.*......	0.2300	chr8

* represents the mismatch position within each off-target site.

**Fig 3:**
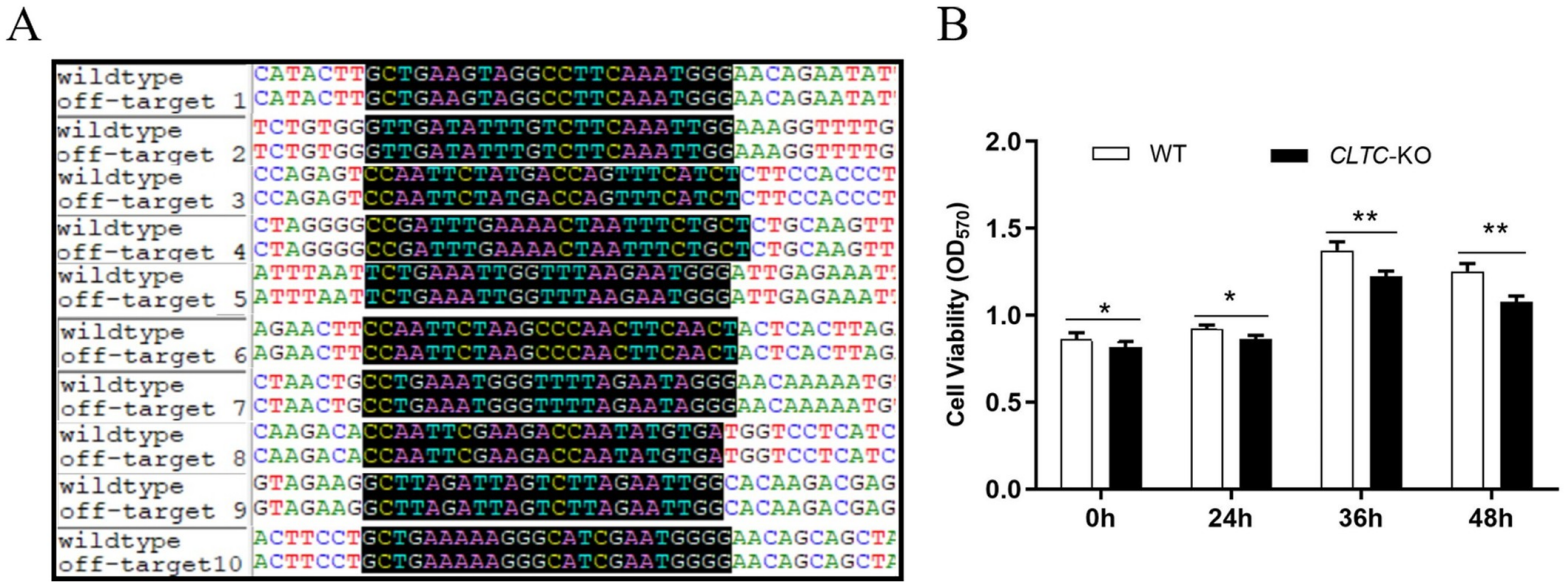
Evalution of off-target effect and cell proliferation activity. **A** Alignment results of top 10 off-target sites sequences in *CLTC* knockout cell line and wild-type cells. **B** *CLTC* knockout caused a decrease in cell proliferation activity. OD_570_ represents the absorbance value detected at 570nm. *p<0.05, **p<0.01.

According to the site information from CRISPOR, primers were designed respectively for each of these sites, and PCR amplification and Sanger sequencing was then performed to compare the sequences of *CLTC* gene knockout cells with the genome of wild-type cell. The results showed that the sequences of 10 targeted sequences of *CLTC* knockout cells were completely consistent with the gene sequences of wild-type cells, the alignment of 10 sites were shown in Fig 3A, which demonstrated that there were no off-target effect at these sites using this sgRNA. Therefore, to some extent, our result preliminarily provided an evaluation of off-target effect for the knockout of *CLTC* gene.

To explore the cell proliferation which is the key foundation to obtain a stable cell line, cell proliferation detection reagent was added to the cell supernatant respectively at 0h, 24h, 36h and 48h after the cells were seeded into 96-well plate. The absorbance value was detected at 570nm. The results showed that the OD570 values of *CLTC*-KO cells were lower than those of wild-type cells at three time points (Fig 3B), which indicated that the knockout of *CLTC* gene caused a slight decrease in the cell proliferation activity.

### *CLTC* knockout caused a significant decrease of SFTSV infection in A549 cells

**Fig 4:**
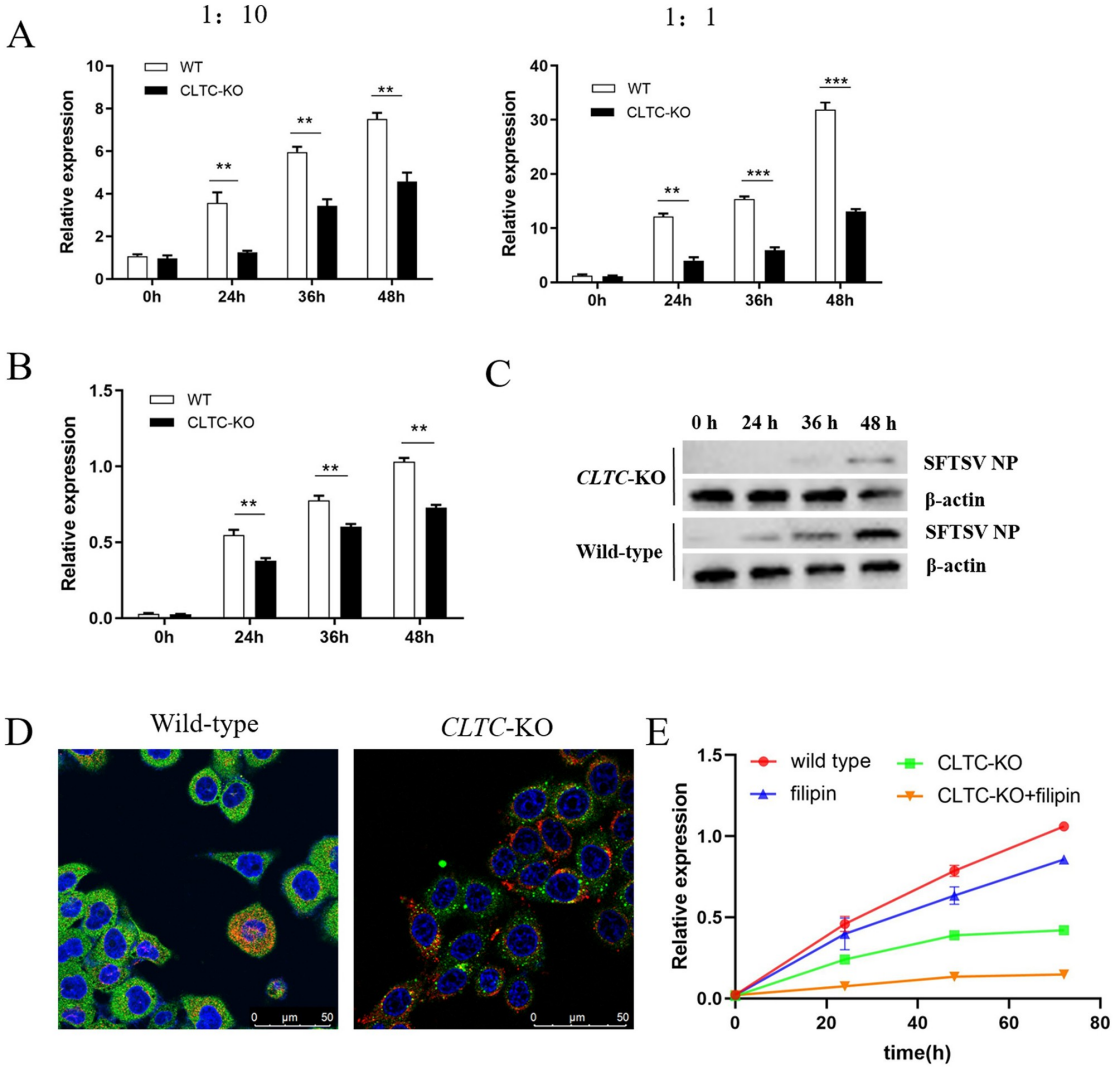
Knockout of *CLTC* caused a decrease infection of SFTSV. **A** Comparison of SFTSV nucleoprotein in supernatant for *CLTC*-KO and wild-type cells. Culture supernatants were collected at 0h, 24h, 36h and 48h after infection, and subjected for ELISA detection for SFTSV nucleoprotein. Supernatants without SFTSV infection was used as negative control. **B** Comparison of SFTSV RNA level in cells for *CLTC*-KO and wild-type. *p<0.05, **p<0.01, ***p<0.005. **C** Replication kinetic of SFTSV nucleoprotein detected by Western blotting analysis. Strips of SFTSV NP in *CLTC*-KO and the wild-type cells were from the same one PVDF membrane. **D** Confocal microscopy for the distribution of SFTSV glycoprotein. *CLTC*-KO and wild-type cells were infected with SFTSV, stained with a mouse monoclonal antibody targeted at SFTSV GP, and a secondary anti-mouse IgG antibody conjugated with Alexa Fluor 488(Invitrogen, A11001). Cell nucleus were stained with DAPI(Beyotime, c1002). **E** Filipin inhibited SFTSV infection. Filipin solution was added to cell culture supernatant, followed by incubation at 37°C for 4 h, rinsed with PBS for three times before SFTSV infection, RT-PCR detection was performed as those described above.

Cells of the wild-type and *CLTC*-KO were infected with SFTSV followed by detection of viral nucleoprotein in the supernant with dilution of 1:1 and 1:10. ELISA analysis showed that SFTSV nucleoprotein expression significantly decreased in *CLTC*-KO cells at three time points in both 1:10 group (Fig 4A, left panel) and1:1 group (Fig 4A, right panel). SFTSV RNA of the infected cells were also isolated followed by RT-PCR detection, which showed that the relative expression of SFTSV RNA significantly decreased in *CLTC*-KO cells compared with that in wild-type cells (Fig 4B).

In addition, Western blotting analysis was used to explore the replication kinetic of SFTSV nucleoprotein which were collected at 0h, 24h, 36h and 48h after infection. The result showed that the expression of SFTSV nucleoprotein increased along with time, both in wild-type and *CLTC*-KO cells. Meanwhile, compared with wild-type cells, the nucleoprotein expression of *CLTC*-KO cells were also significant decreased at three time points (Fig 4C), which was consistent with the result of RT-PCR and ELISA described above.

To verify this phenomenon in *CLTC* knockdown cells, we selected the *CLTC* knockdown cell line obtained in Fig 2A with Sanger sequencing results shown in S1A Fig. Then, we performed SFTSV infection using this cell line and RT-PCR to detect SFTSV RNA in the infected cells, and the result showed that the knockdown of CLTC protein can still lead to a significant decrease of SFTSV infection at 24h, 36h and 48h post infection (S1B Fig). Therefore, we, for the first time, using cell model with CLTC protein deletion, confirmed that CLTC protein deletion did not completely block SFTSV infection.

### SFTSV enter host cells using both clathrin-dependent and independent endocytosis

In order to preliminarily explore the mechanism of blocked SFTSV infection, we performed a confocal microscopic analysis at 24h after SFTSV infection in wild-type and *CLTC*-knockout cells. The result showed a stronger fluorescence intensity and a uniform distribution of SFTSV glycoprotein in the cytoplasm of wild-type cells, while it showed a weaker fluorescence intensity of glycoproteins in the *CLTC*-knockout cells, as shown in Fig 4D. These results, to some extent, may provide a clue that the deletion of clathrin may block the entry process of SFTSV into cytoplasm from cell membrane.

To further investigate which endocytosis mode except CME could be used during SFTSV entry, Filipin, a lipid raft inhibitor and EIPA, a macropinocytosis inhibitor were added to the cell culture supernatant, respectively, followed by SFTSV infection and RT-PCR experiment to detect SFTSV RNA at different time points post infection. Our results showed that SFTSV infection was significantly decreased at 24h, 48h and 72h in cells with Filipin treatment compared with the wild-type cells, and more significantly, it was decreased in Filipin treated *CLTC*-KO cells (Fig 4E), while it was not significantly decreased in wild-type cells with EIPA treatment, as shown in S2 Fig. This result demonstrated that lipid raft could also be used by SFTSV to enter host cells, and since the infection was almost completely blocked in this group, CME and lipid raft mediated entry may be the major two endocytosis for SFTSV. In conclusion, using *CLTC*-KO cell line, our study demonstrated that SFTSV may enter host cells via both clathrin dependent and independent endocytosis.

## Discussion

Clathrin plays a crucial role in the process of viral endocytosis. There have been many functional studies on clathrin [19, 20], but these researches have certain limitations, because the use of clathrin inhibitors or gene knockdown methods could not delete the protein completely [21–23]. Since *CLTC* gene knockout has not been reported before, this study attempted to establish *CLTC* gene knockout cell line to find direct evidence for the functional study of clathrin.

Lentivirus-based CRISPR gene editing technology can integrate the sgRNA sequence into the genome of the cell, and stably and continuously express with cell proliferation [24, 25]. Therefore, compared with plasmid transfection, this method can obtain more stable cell lines [14, 26]. In addition, due to the wider spectrum of cells infected by lentivirus and the ability to avoid cytotoxic effects, the application of lentivirus-based CRISPR gene editing technology is becoming more and more widespread. However, there are certain defects for this method, for example, the integrated sequence may have possibility to cause gene mutagenesis and carcinogenesis, and the process of lentivirus packaging is time-consuming, cumbersome, and requires high technical requirements for laboratory personnel.

In this study, Sanger sequencing, Western blotting and immunofluorescence analysis were used to verify the knockout effect of CLTC protein at DNA and protein levels, and the off-target effect of sgRNA was evaluated by sequencing the 10 sites that were most likely to have off-target effect, which ensured the reliability of this gene knockout cell line and provided a methodological reference for researchers to construct *CLTC* knockout cells. This cell line we established has been passaged for 34 times, and there was still no CLTC protein expression.

In fact, when CRISPR/Cas9 is used for gene knockout, the probability of constructing a homozygous knockout cell line is very low, because a homozygous cell line requires that the knockout status of two homologous chromosomes be exactly the same, while the host cell repair the incision in a random manner. Although the cell lines established in this study were not homozygous, the results of Western blot and Immunofluorescence assay showed that CLTC protein was no longer expressed, which indicated that heterozygous gene knockout cell lines can also be used for protein functional studies.

This research found that *CLTC* knockout caused a significant decrease in SFTSV infection, which supports the conclusion that SFTSV enters host cells via clathrin dependent endocytosis. However, it was noted that, after *CLTC* knockout, SFTSV could still infect A549 cells in a small amount, indicating that CME is not the only way of SFTSV entry. By completely deleting the CLTC protein, our study provided direct evidence that SFTS virus can enter host cells without clathrin expression. However, we cannot rule out the possibility of delayed infection in CLTC-KO cells rather than a reduction in infection. Besides, our research demonstrated that macropinocytosis inhibitor EIPA could not reduce SFTSV infection, but lipid raft inhibitor Filipin could significantly reduce SFTSV infection, which indicated that SFTSV could use lipid raft mediated endocytosis to enter host cells.

There are some certain limitations in this research. For example, only one sgRNA targeting the exon of *CLTC* was designed in this study, while two or more sgRNAs were used in the knockout of other genes whose knockout efficiency may be higher [27, 28]. Besides, the method in which only top 10 sites were detected for the off-target effect, was to some extent one-sided, and the evaluation of off-taget effect based on whole genome sequencing was more comprehensive and convincing. In addition, a 96-well plate limited dilution method was used to obtain single cloned cells, which was time-consuming and labor-intensive to obtain a monoclonal cells and it would be more convenient and accurate if Flow cytometry was used to seed single cells into each well of 96-well plates.

In conclusion, this study for the first time, established a *CLTC* gene knockout cell line, which provided a technical reference for researchers to establish *CLTC* knockout cell line. In addition, this research confirmed that SFTSV could infect cells without clathrin expression despite a significant decrease, and lipid raft mediated endocytosis, as a clathrin-independent pathway, could be another key mode for SFTSV entry. Therefore, our research laid a solid foundation for study on the mechanism of endocytosis and provide theoretical support for the discovery of drug targets for SFTSV.

## Supporting information

S1 Fig Knockdown of CLTC protein inhibited SFTSV infection. (TIF)

S2 Fig Macropinocytosis inhibitor EIPA did not reduce SFTSV infection. (TIF)

S1 Raw images (PDF)
