# Supplementary figures and images for: Knockout of CLTC gene reduces but not completely block SFTSV infection
Source: PLoS One. 2023 Aug 25;18(8):e0285673. doi: 10.1371/journal.pone.0285673 (PMC10456188; doi:10.1371/journal.pone.0285673)

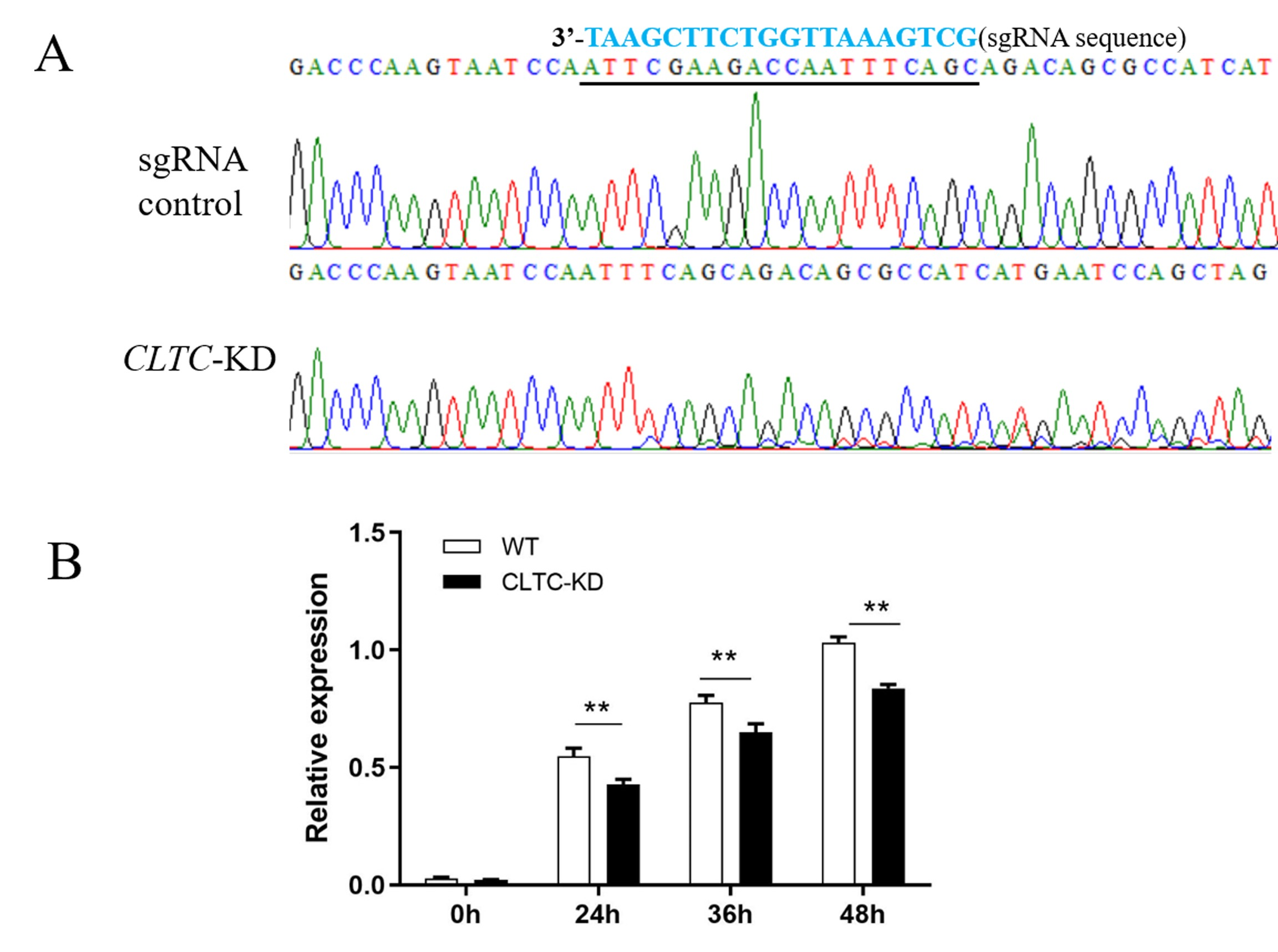

Supplement: S1 Fig — (TIF) [file pone.0285673.s001.tif]

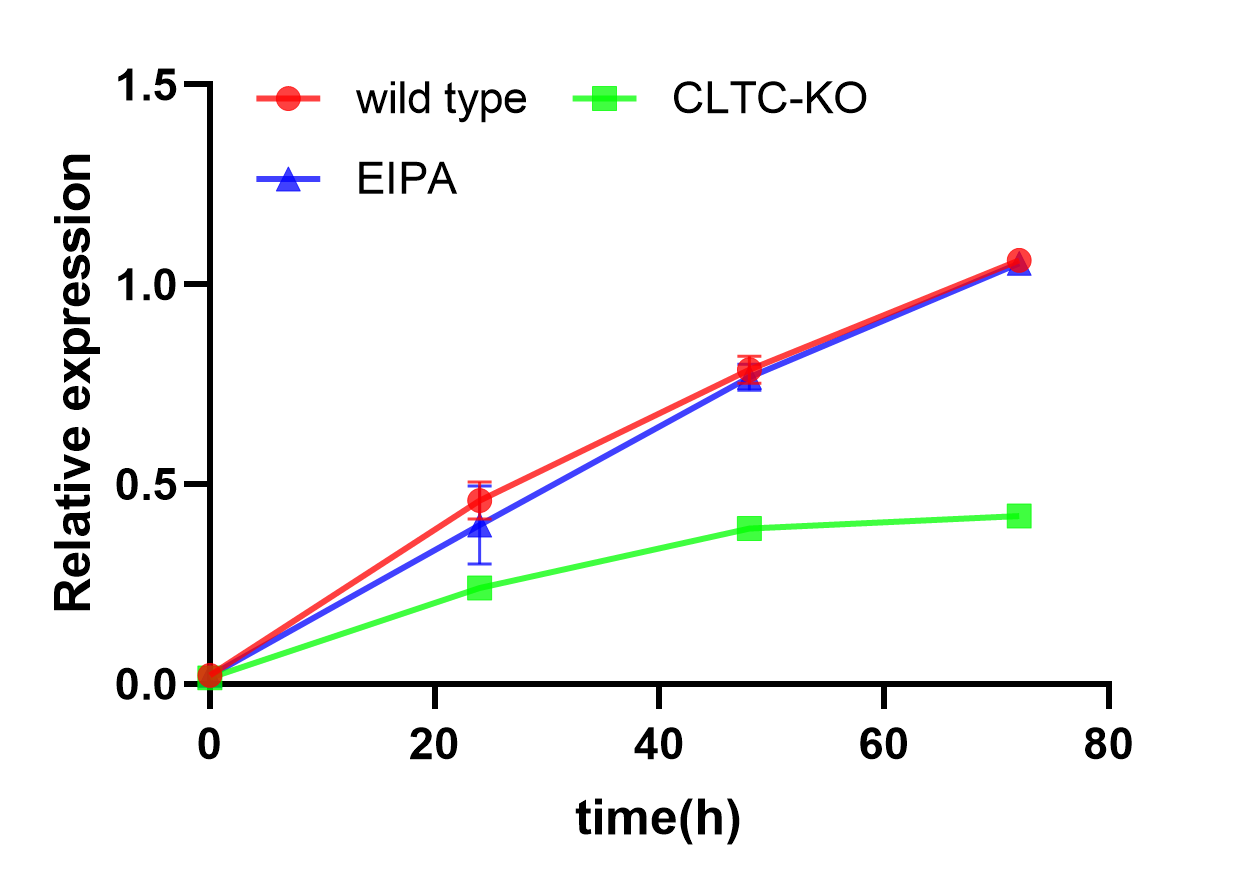

Supplement: S2 Fig — (TIF) [file pone.0285673.s002.tif]

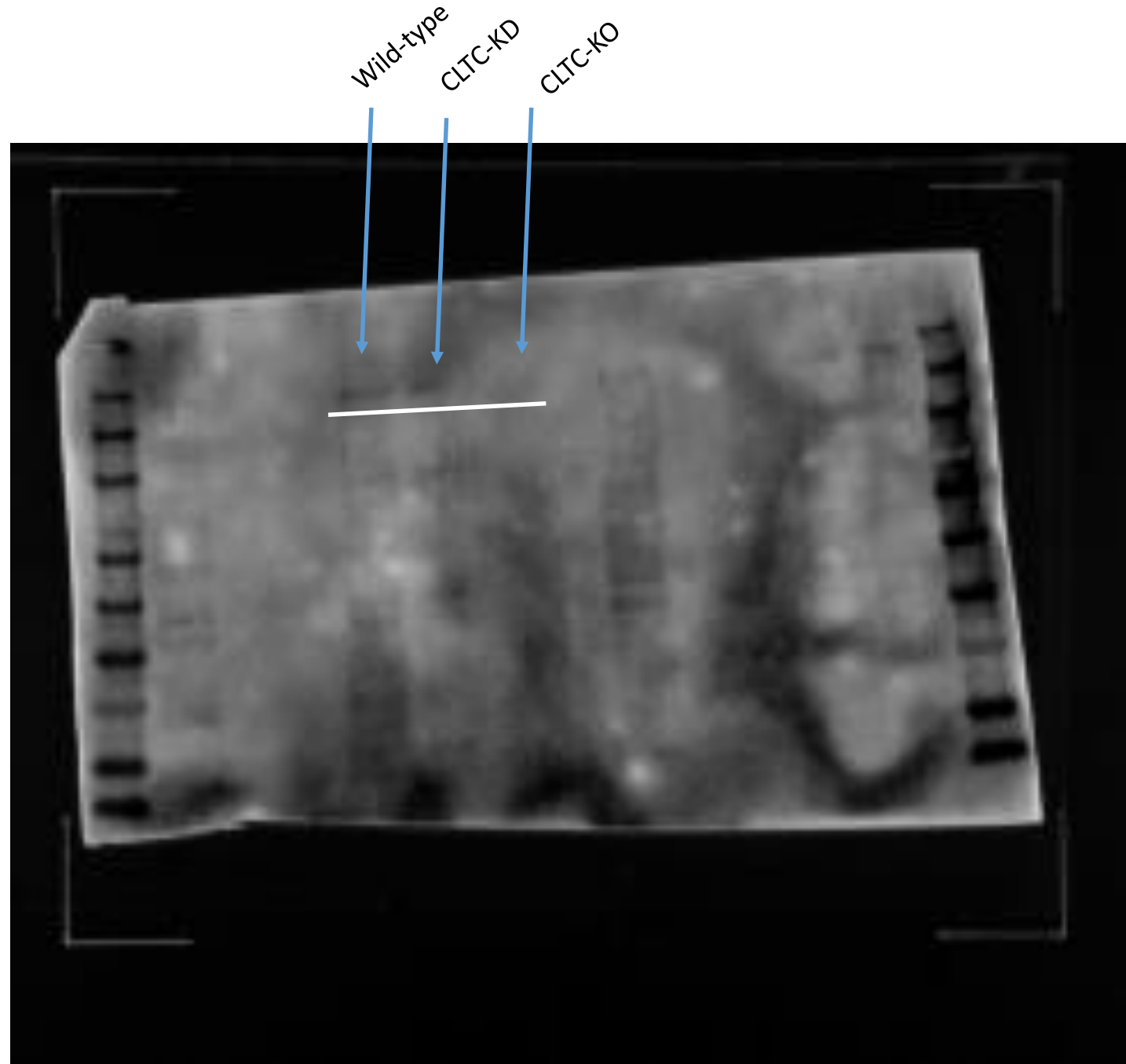

Figure 2A CLTC

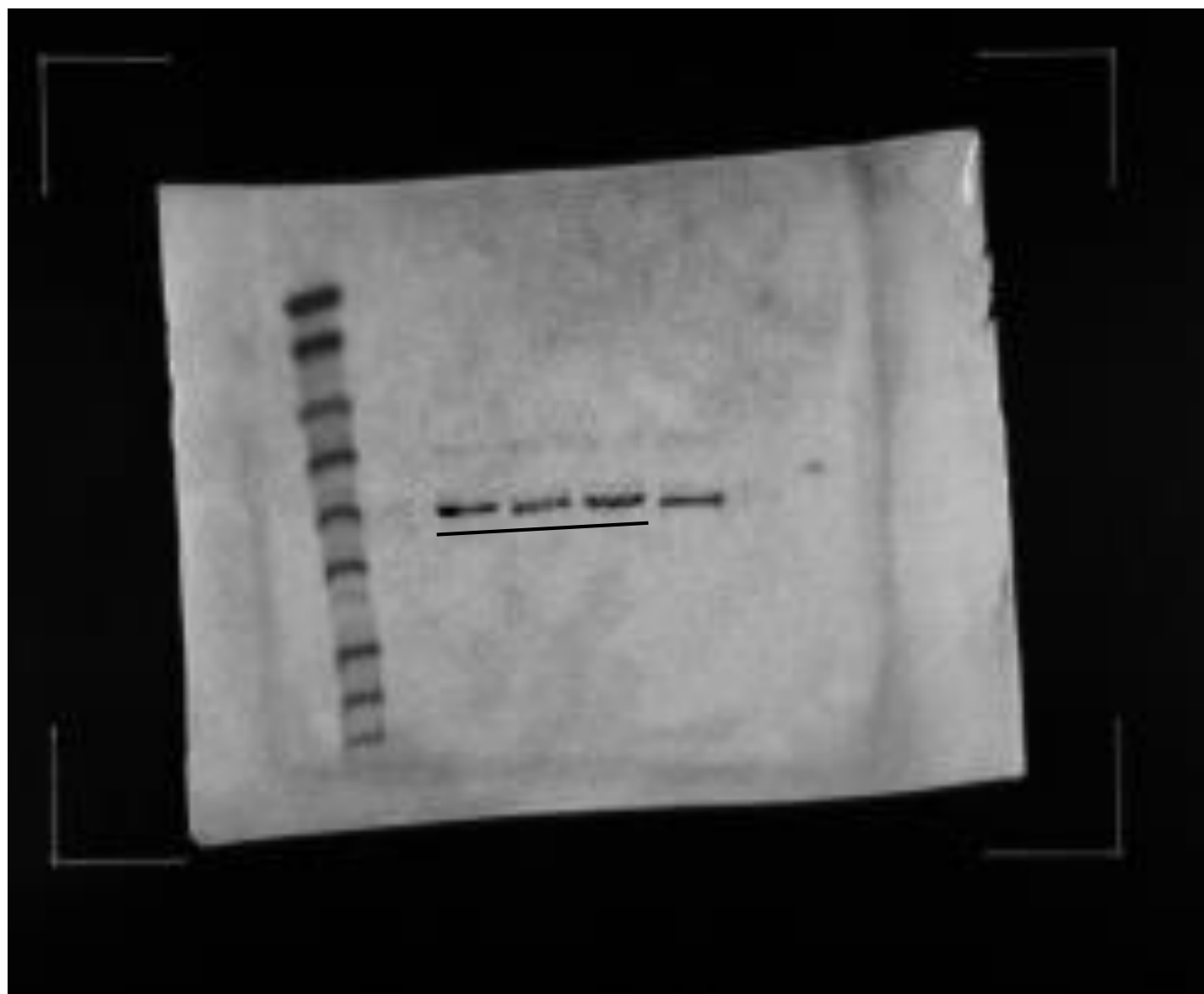

Figure 2A  $\beta$ -actin

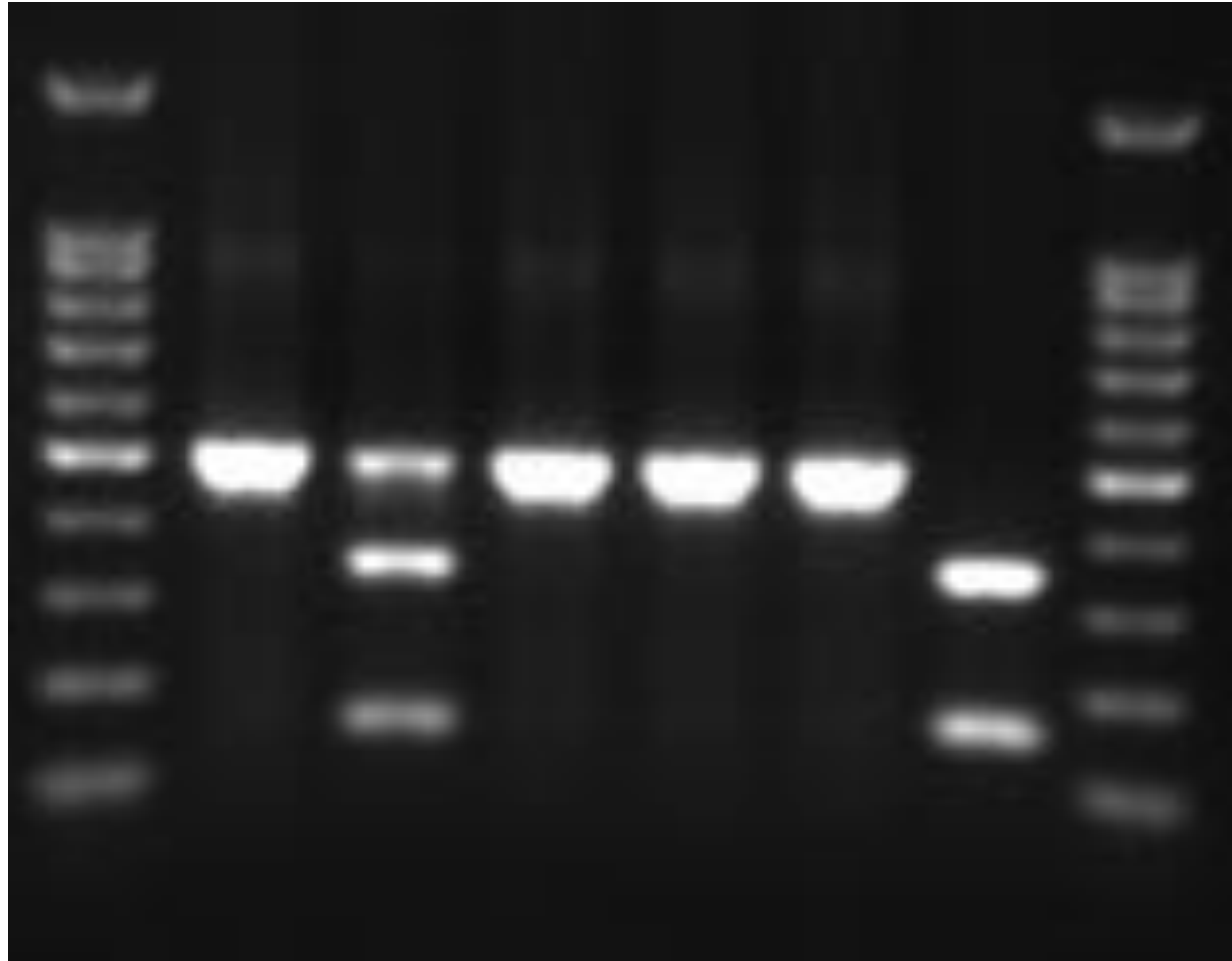

Figure 2D

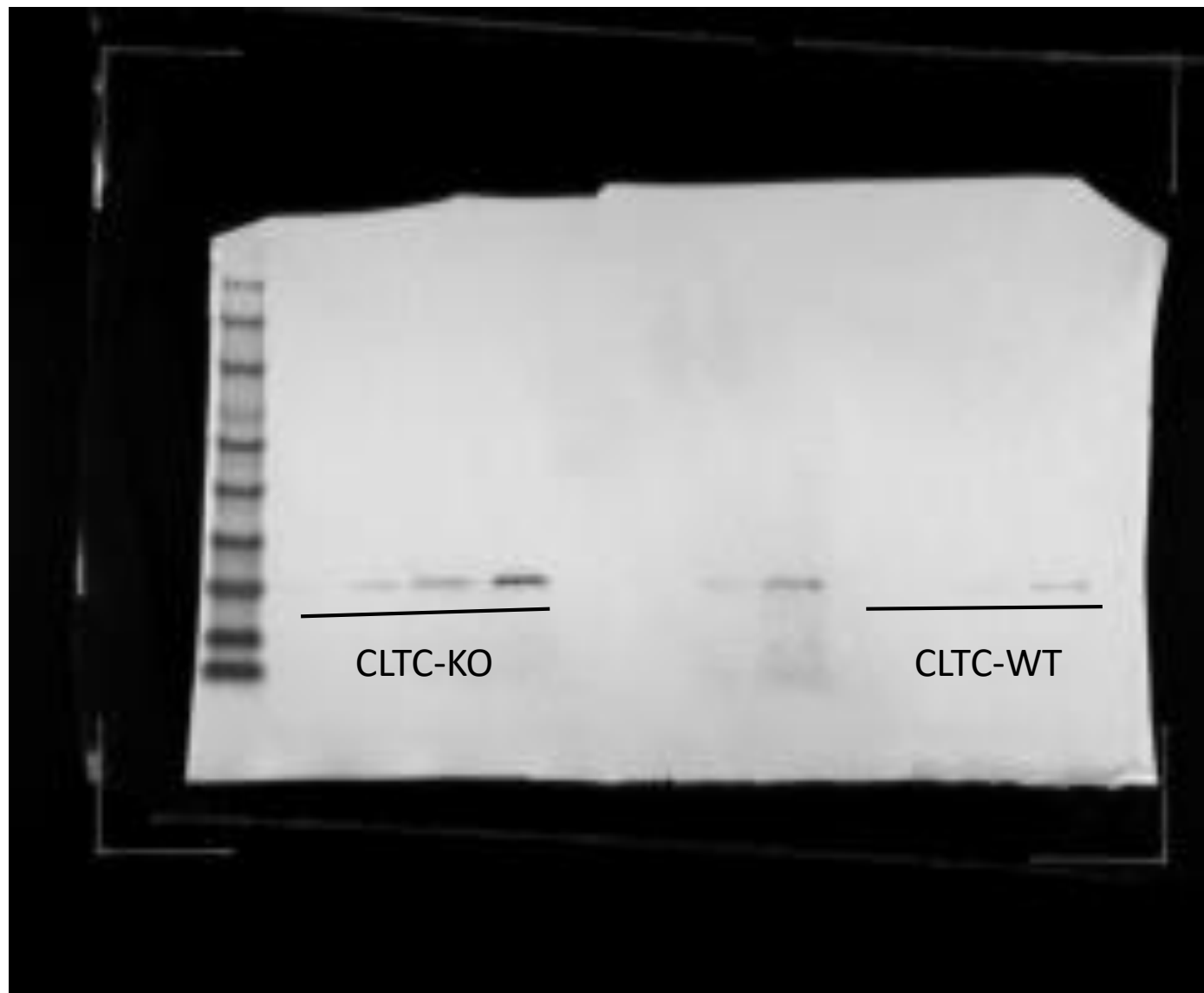

Figure 4D SFTSV-NP

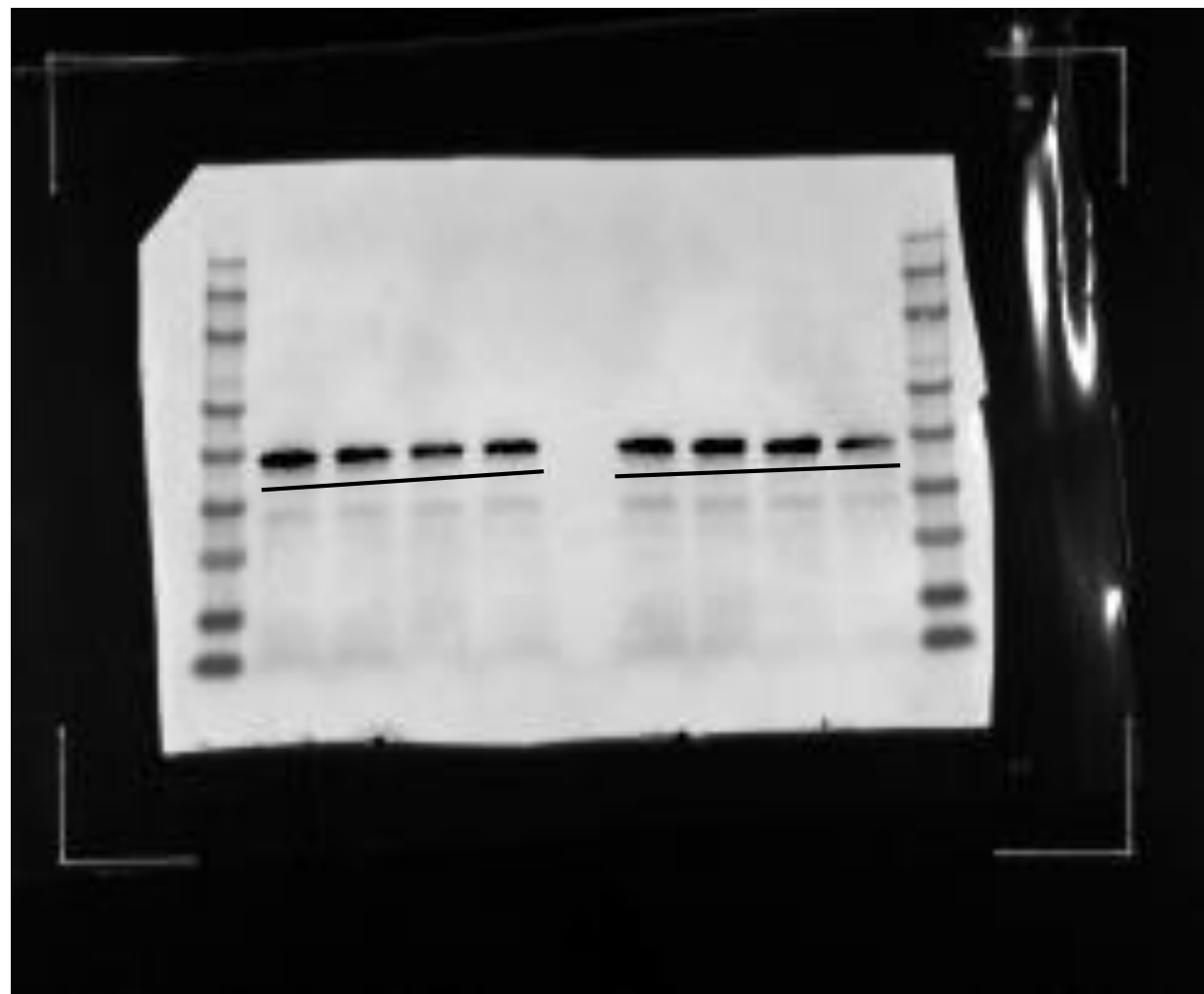

Figure 4D  $\beta$ -actin

Supplement: S1 Raw images — (PDF) [file pone.0285673.s003.pdf]
